# Spatiotemporal distribution and predictors of tuberculosis incidence in Morocco

**DOI:** 10.1186/s40249-018-0429-0

**Published:** 2018-06-07

**Authors:** Mina Sadeq, Jamal Eddine Bourkadi

**Affiliations:** 1 Environmental Epidemiology Unit, National Institute of Hygiene. Ministry of Health, Rabat, Morocco; 2 University Hospital Center. Moulay Youssef Hospital, Rabat, Morocco; 3 Faculty of Medicine and Pharmacy, University Mohammed V, Rabat, Morocco

**Keywords:** TB, Meteorological data, Prefecture/province, AIDS, Population density, Morocco

## Abstract

**Background:**

Tuberculosis (TB) is a major health problem in Morocco. This study aims at examining trends in TB in Morocco and identifying TB spatial clusters and TB-associated predictors.

**Method:**

Country-level surveillance data was exploited. Kendall’s correlation test was used to examine trends and an exploratory spatial data analysis was conducted to assess the global and local patterns of spatial autocorrelation in TB rates (Moran’s *I* and local indicator of spatial association [LISA]) at the prefecture/province level. Covariates including living in a prefecture versus living in a province, annual rainfall, annual mean temperature, population density, and AIDS incidence were controlled. An ordinary least squares regression was thus performed and both spatial dependence and heteroscedasticity were assessed.

**Results:**

A decrease in TB incidence rate was seen between 1995 and 2014 (Kendall’s tau b = − 0.72; *P* <  0.0001). However, while the period between 2005 and 2014 (10 last years) was considered, TB rate remained stable and as high as 84 per 100 000 population per year (95% *CI*: 83.7–84.3). The highest incidence rates were seen in Tanger-Assilah, Fez, Tetouen-M’diq Fnidaq, Inezgane Ait Melleoul, and Casablanca. From 2005 to 2014, while TB incidence rate was stable in Fez (*P* = 0.500), Tetouen-M’diq Fnidaq (*P* = 0.300), Casablanca (*P* = 0.500), Mohammadia (*P* = 0.146), Al Hoceima (*P* = 0.364), and Guelmim (*P* = 0.242), an increase in TB incidence rate was seen in Tanger-Assilah (Kendall’s tau = 0.49; *P* = 0.023) and a decrease in Salé (Kendall’s tau b = − 0,54; *P* = 0.014) and Inezgane-Ait Melloul (Kendall’s tau b = − 0,67; *P* = 0.0023). TB is strongly clustered in space (*P*-values of Moran’s *I* <  0.01). Two distinct spatial regimes that affect TB spatial clustering were identified (east and west). In the east, both annual rainfall (*P* = 0.003) and AIDS (*P* = 0.0002) exert a statistically significant effect on TB rate. In the west, only the living area (prefecture versus province) was associated with TB rate (*P* = 0.048).

**Conclusions:**

New information on TB incidence and TB-related predictors was provided to decision-making and to further pertinent research. Association between annual rainfall and TB may be of interest to be explored elsewhere.

**Electronic supplementary material:**

The online version of this article (10.1186/s40249-018-0429-0) contains supplementary material, which is available to authorized users.

## Multilingual abstract

Please see Additional file 1 for translations of the abstract into the five official working languages of the United Nations.

## Background

Tuberculosis (TB) is one of the top 10 causes of death worldwide [1]. In 2015, 10.4 million people around the world fell ill from TB and a total of 1.8 million died from this disease. Over 95% of deaths from TB occur in low and middle income countries [1]. In Morocco, TB remains a major public health problem in spite of the efforts of the Ministry of health (MH) to alleviate it [2]. In 2015, 30 636 cases were reported; a total of 656 cases died from TB [3].

A national TB program was set at the end of the seventies to prevent, control, and eventually eliminate TB from Morocco. Standardized treatment regimens are provided for free [4]. Two reference national laboratories provide testing for TB infection. In 2004, Morocco managed to reach the WHO objectives related to TB diagnosis and treatment [2]. Thus, in 2015, 83% of the cases were detected, 85% were treated for TB [2]. However, TB incidence did not seem to decrease in Morocco. The recent statistics showed that TB incidence in Morocco was as high as 89 per 100 000 population in 2015 [2].

More may need to be explored about TB in Morocco. Studies on spatial clusters of TB incidence that would have given better understanding where interventions are most required are lacking in Morocco. On the other hand, it is thought that TB prevails in prefectures rather than in provinces, and that the population density is a risk factor of TB in Morocco. Such claims require further research. Association between TB incidence and meteorological factors has been cited elsewhere [5–7], but has not been explored in Morocco yet. Cases of AIDS/ HIV are more vulnerable to TB infection. Including AIDS/HIV incidence as a covariate in a regression model would best predict TB incidence in Morocco.

This work aimed at, first, examining trends in TB incidence rate in Morocco; second, examining spatial clustering/clusters of TB incidence at the province/prefecture level; third, exploring non-spatial and spatial correlation between TB and some covariates in order to specify a model that would best predict TB in Morocco. Potential predictors are living in a prefecture versus living in a province, population density, AIDS incidence, and meteorological factors, i.e., annual rainfall and annual mean temperature. Diagnostics for spatial dependence and spatial heterogeneity were performed. Spatial study was limited to the last four years (i.e., 2011 to 2014) for a reason cited in “Results” section.

## Methods

### Geographical data, study area/population, and population density by year

A polygon shapefile map of Morocco comprising 59 provinces/prefectures, developed for a previous study [8], was used. The process of georeferencing, digitalizing, and combining some provinces/prefectures is described elsewhere [8]. Data on population size by province/prefecture were obtained from “Santé en Chiffres” files that were made available by the Service of Studies in Health and Health Information-Ministry of Health (SSHHI-MH) [9]. The total population size was 32 187 000 inhabitants in 2011, it was 33 848 000 inhabitants in 2014. For each year under study, the population density by province/prefecture was calculated; thus, the population in a province/prefecture was divided by the size of that province/prefecture.

### TB data by year

Data on both new cases and incidence of TB by province/prefecture were obtained from “Santé en Chiffres” files made available by SSHHI-MH [9]. The raw incidence rates of TB by province/prefecture were calculated; outliers were looked for. Thus box map was displayed to check for variance instability of the raw rates. GeoDa software version 1.6.7.9, March 2015, developed by Luc Anselin (ASU, GeoDa Center for Geospatial Analysis and Computation, Arizona, USA), was used for these purposes. This was performed for each year under study.

### Data on potential predictors of TB

#### Meteorological data by year

A centroid for each polygon that represents a province/prefecture was created and its GWS84 coordinates were determined. Those coordinates were used to get meteorological data (annual rainfall and annual mean temperature) by province/prefecture. Climate monitoring data were obtained from the Global Climate Monitor [10] made available under the Open Database License. This was performed for each year under study. The QGIS software version 2.0.1 ‘Dufour’ (Free Software Foundation, Inc., Boston, USA) was used.

#### Spatial regime

Box maps of raw TB incidence rate were examined first and foremost. High TB rates were seen in the west of Morocco, low TB rates in the remaining part of the country, and this may suggest the possible presence of spatial heterogeneity in the form of spatial regimes. Thus, it was hypothesized that TB predictors may exert a different effect across the west and east of Morocco. In this study, these spatial regimes, i.e., west versus east, were identified as shown in Fig. 2, and were evaluated as a dummy variable in the statistical spatial analyses. They will be incorporated into the multivariate analyses that adjust for spatial heterogeneity.

#### HIV/AIDS by year

Data on HIV are not available and only those on AIDS incidence (by province/prefecture) of 2008 and 2009 are [9], and this imposed a constraint as to the multivariate regression. To deal with this, it was first opted for data on AIDS of 2009 and it was checked whether the other potential predictors affect TB in the years between 2011 and 2014 and in 2009, similarly. If it is the case, AIDS rate can then be incorporated as an additional covariate in the multivariate regression to draw conclusions about the effect of this variable on TB incidence.

### Statistical analysis

#### Trends in TB

An approximate two-sided Kendall’s rank correlation test was conducted to examine variation in TB incidence from 1995 to 2014 (20 years) and from 2005 to 2014 (10 years); the *P*-values and size effects of which are provided. An annual Poisson incidence rate estimate of TB and a Poisson rate confidence interval were also provided. The incidence rate is estimated as the number of events observed divided by the time at risk of event during the observation period.

A Kendall’s rank test was performed to evaluate variation in the incidence rate from 2011 to 2014 in selected prefectures and provinces. Statistics were calculated in exact form.

These statistical methods were conducted using the StatsDirect statistical software version 3.0.194 (StatsDirect Ltd., Cheshire, UK).

#### Global spatial clustering and LISA clusters of TB

The exploratory spatial data analysis approach [11–16] was used to examine global and local patterns of spatial autocorrelation in TB rates and in covariates. A contiguity raw standardized weight file was created. Queen contiguity, which defines spatial neighbours as those provinces/prefectures with shared borders and vertices, was chosen. Thus, the global univariate Moran’s *I* statistic was examined. A positive and significant Moran’s *I* indicates clustering in space of similar TB rates. The local indicators of spatial association (LISA), which show the presence or absence of significant spatial clusters or outliers, was also examined. GeoDa software was used to perform these spatial analyses.

#### Specifying a regression model of TB

Global clustering of potential predictors was examined first and foremost. GeoDa was used to perform Moran’s *I* test. Then, potential multicollinearity and linear correlation between TB and predictors were analysed. StastDirect was used to perform Kendall’s rank correlation tests. Then, GeoDa was again used to perform bivariate Moran’s *I* test to examine bivariate LISA between TB and covariates. In addition, an ordinary least squares (OLS) regression analysis that took into account the previously identified spatial regimes was conducted. GeoDa was used for this purpose. Multicollinearity condition number, normality (Jarque-Bera test), spatial dependence for weight matrix (row-standardized weights and Lagrange multiplier tests), and spatial heteroskedasticity (Breusch-Pagan test and Koenker-Bassett test) were all assessed. Then, the stability of predictors effect across regimes was evaluated. GeoDaSpace (ASU, GeoDa Center for Geospatial Analysis and Computation, Arizona, USA) was used to perform a Chow test.

## Results

### Trend in TB incidence in Morocco from 1995 to 2014 and from 2005 to 2014

**Fig. 1:**
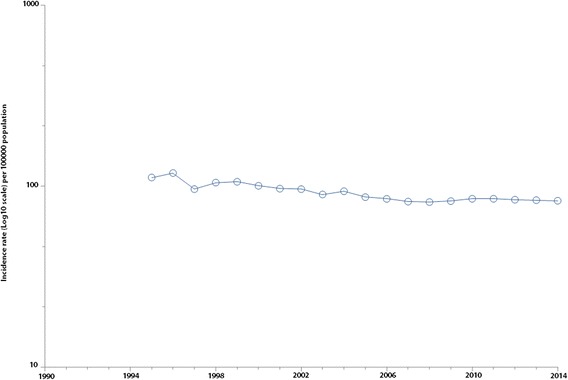
Incidence rate (Log10 scale) of tuberculosis, Morocco, 1995–2014. This figure depicts secular trends of tuberculosis in Morocco from 1995 to 2014. A decrease was shown during this 20-year period (Kendall’s tau = − 0.72; *P* = 0.0001). TB incidence rate remained relatively stable at 85.3 per 100 000 population from 2005 to 2014

A decrease in TB incidence rate was seen from 1995 to 2014 in Morocco (Fig. 1) (Kendall’s tau b = − 0,72 *P* < 0.0001). The mean estimate of new TB cases (all forms confounded) was 27 642. A maximum number of 31 771 cases was reported in 1996, a minimum number of 25 473 cases in 2008. While the period between 2005 and 2014 was considered, TB incidence rate was stable (Kendall’s tau = − 0.16; *P* = 0.242). During this 10-year period, TB incidence rate mean was 85.3 per 100 000 population, *SD* = 3.5. The Poisson annual incidence rate estimate was 84 per 100 000 population per year at 95% *CI*: 83.7–84.3.

### Descriptive analysis and trends in TB incidence in prefectures and provinces

**Fig. 2:**
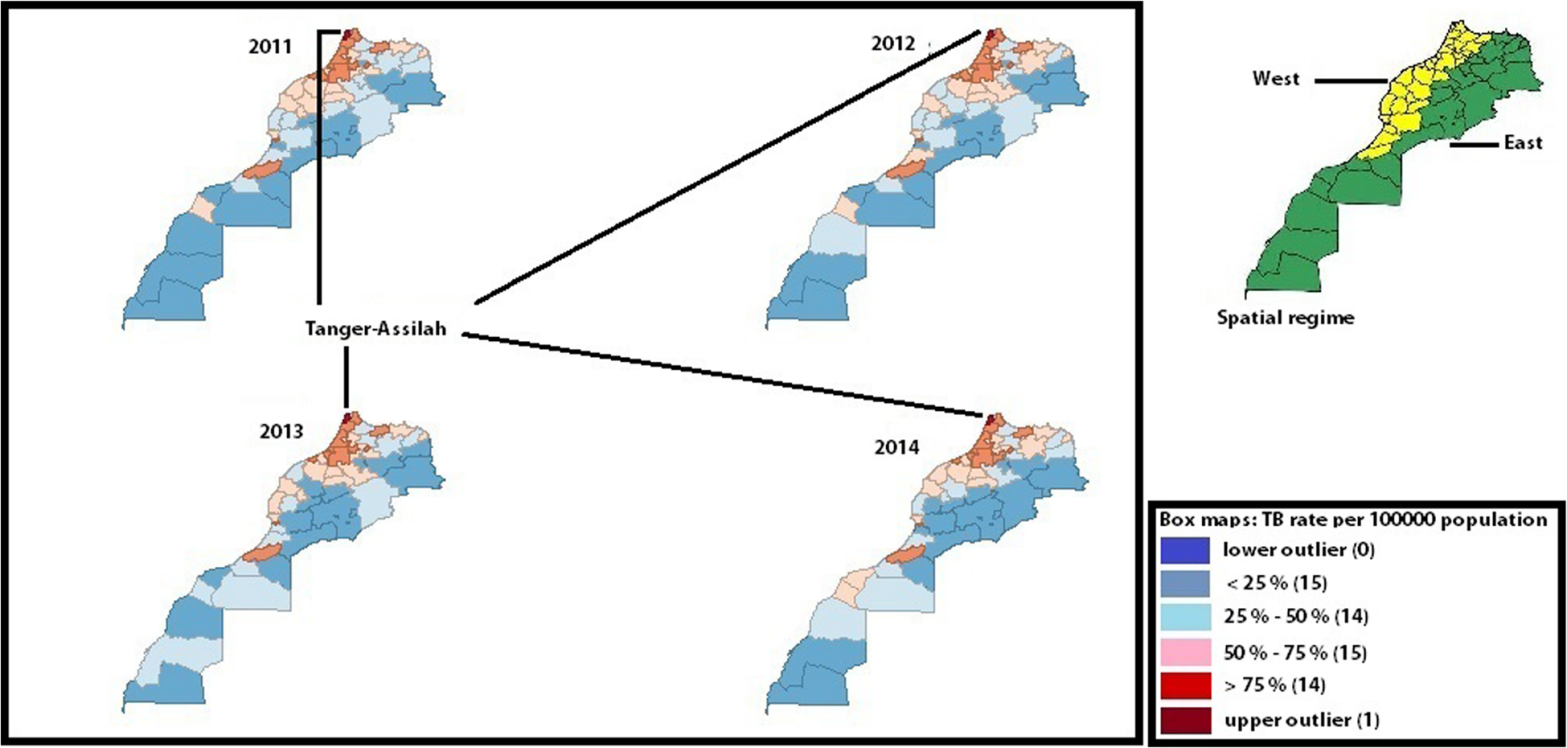
TB raw rate box maps (using 1.5 as hinge). 2011–2014. Morocco. Examining raw TB rate box maps revealed that more than 25% of data were located in the west part of the country where high TB levels were shown (in yellow) while low TB levels were seen in the west part of the country (in green), and this identified two distinctive spatial regimes

**Fig. 3:**
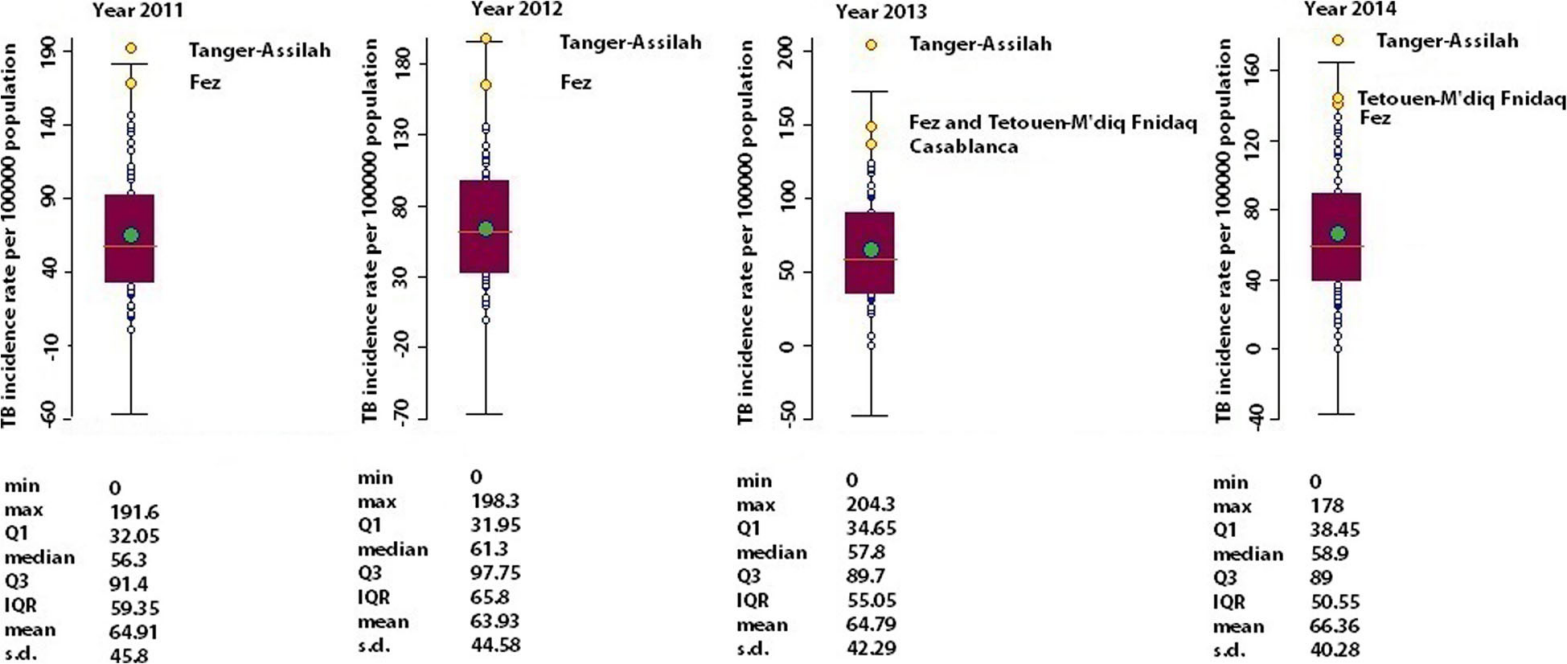
Descriptive analysis of TB incidence between 2011 and 2014. Morocco. The highest incidence rate, a unique outlier, was shown in the prefecture of Tanger-Assilah. High TB rates were also shown in the prefectures of Fez, Tetouen-M’diq Fnidaq, and Casablanca

**Fig. 4:**
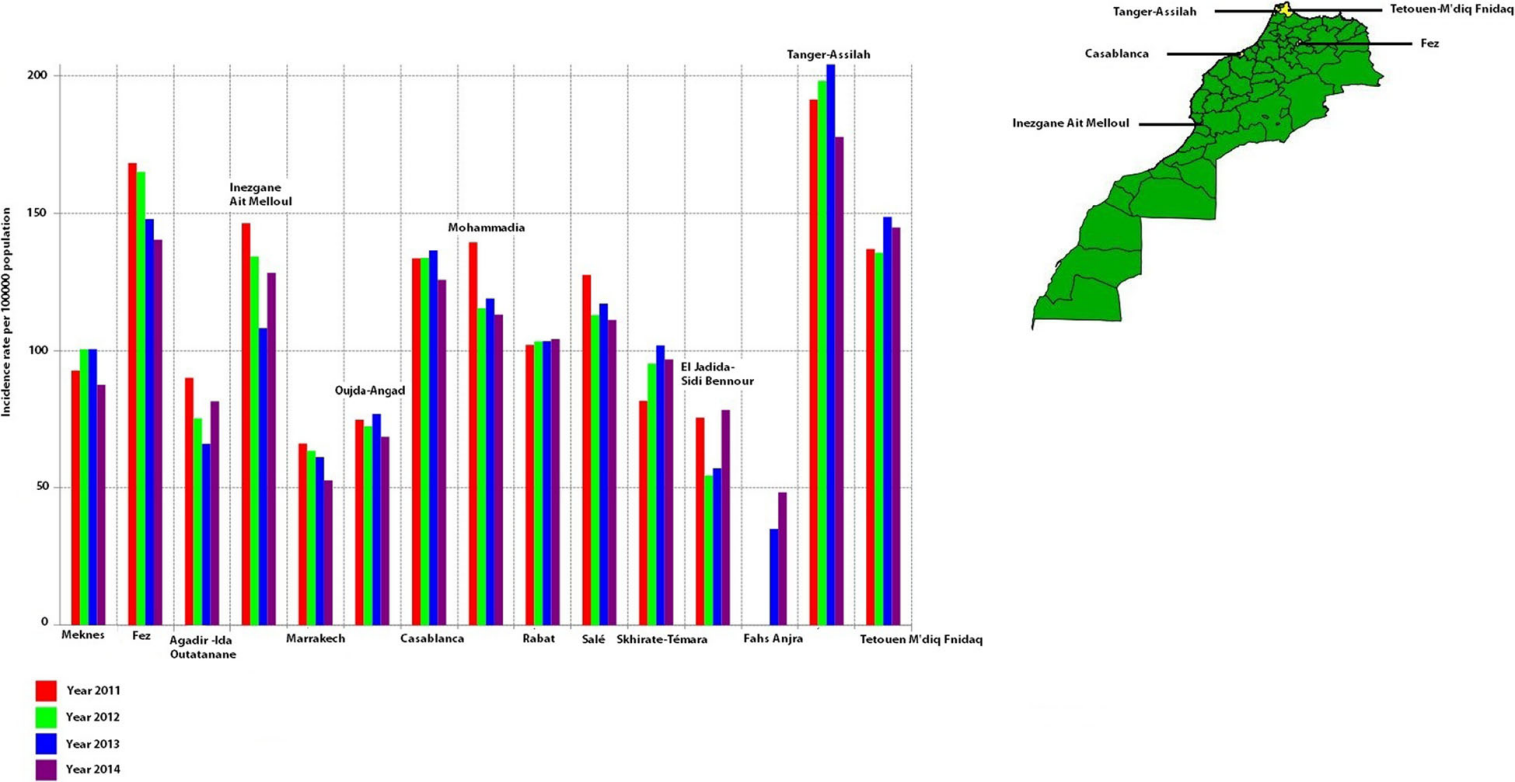
TB incidence rate by year (2011–2014), prefectures, Morocco. Legend: This figure depicts variation of TB incidence rate in the prefectures of Morocco. During the period from 2011 to 2014, the Highest TB incidence rates were seen in Tanger-Assilah, Fez, Tetouen-M’diq Fnidaq, Inezgane-Ait Melloul and Casablanca, all showed in the map. We looked more closely at TB variation in these prefectures during the period from 2005 to 2014. An increase in TB incidence rate was seen in Tanger-Assilah (Kendall’s tau = 0.49; *P* = 0.023) while a decrease was seen in Inezgane-Ait Melloul (Kendall’s tau b = − 0,67; *P* = 0.0023). TB incidence rate was stable in Fez (*P* = 0.5), Tetouen-M’diq Fnidaq (*P* = 0.300), and Casablanca (*P* = 0.5)

The highest incidence rate was shown in the prefecture of Tanger-Assilah, identified as the unique outlier in Figs. 2 and 3. Figure 4 shows TB incidence rates in all the prefectures of Morocco. High TB rates were seen in Tanger-Assilah, Fez, Tetouen-M’diq Fnidaq, Inezgane-Ait Melloul, Casablanca, Mohammadia, and Salé. The respective means (expressed per 100 000 population) and standard deviation for the period between 2005 and 2014 were 188 ± 12, 156 ± 12, 133 ± 13, 153 ± 26, 134 ± 13, 122 ± 10, and 125 ± 8. As to provinces, high TB incidence rates were seen in Al Hoceima (119 ± 7), Guelmim (104 ± 19), Khemissat, and Larache. From 2005 to 2014, while TB incidence rate was stable in Fez (*P* = 0.5), Tetouen-M’diq Fnidaq (*P* = 0.300), Casablanca (*P* = 0.5), Mohammadia (*P* = 0.146), Al Hoceima (*P* = 0.364), and Guelmim (*P* = 0.242), an increase in TB incidence rate was seen in Tanger-Assilah (Kendall’s tau = 0.49; *P* = 0.023) and a decrease in Salé (Kendall’s tau b = − 0,54; *P* = 0.014) and Inezgane-Ait Melloul (Kendall’s tau b = − 0,67; *P* = 0.0023).

### Global spatial clustering and LISA of TB incidence between 2011 and 2014

**Fig. 5:**
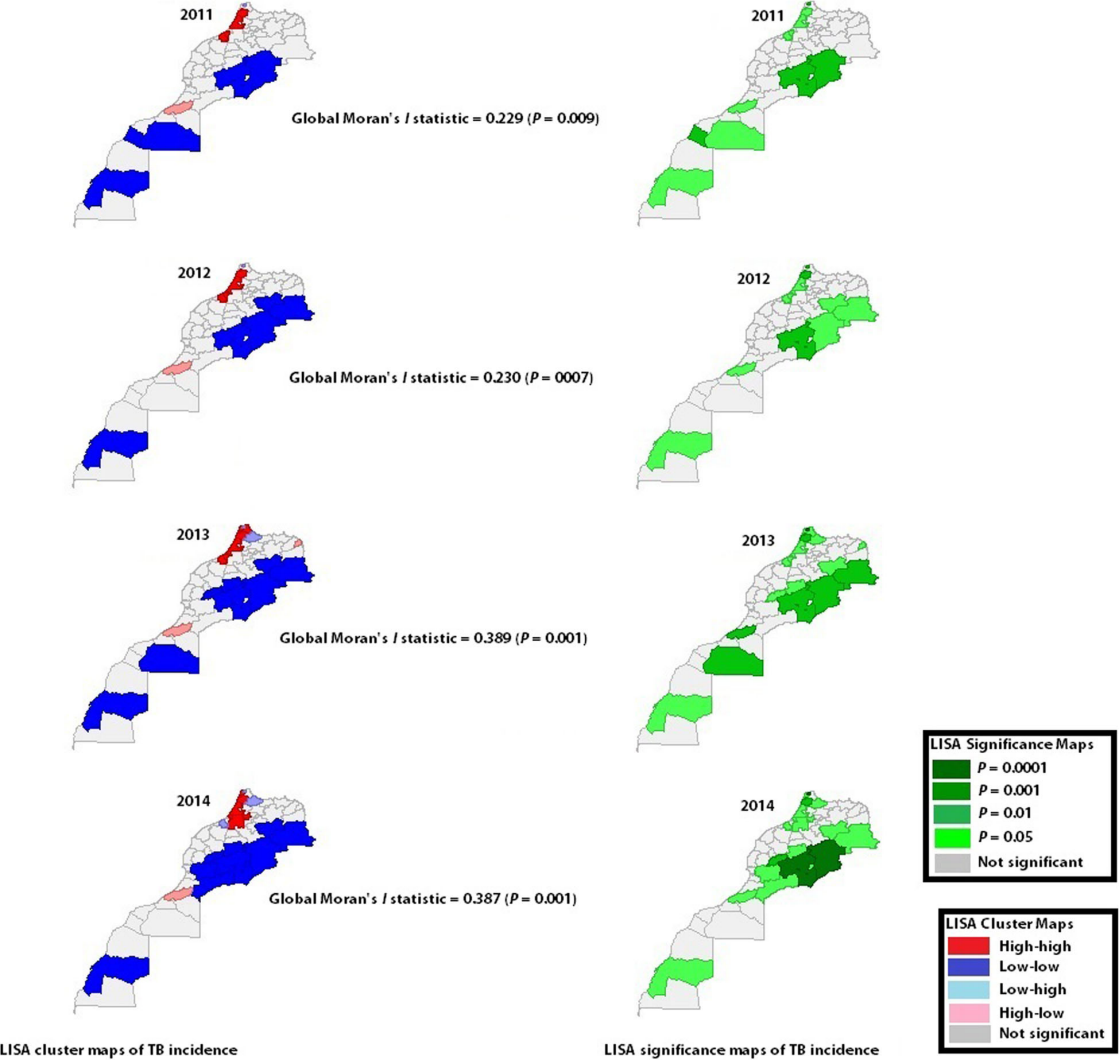
LISA cluster maps and LISA significance maps of TB incidence, Morocco, 2011–2014. LISA indicates the presence or absence of significant spatial clusters or outliers for each province/prefecture. The province of Larache and the province of Kenitra formed spatial clusters of high TB incidence from 2011 to 2014, the prefecture of Skhirate-Témara and the province of Benslimane from 2011 to 2013, the prefecture of Salé from 2012 to 2014, and the prefecture of Tanger-Assilah from 2013 to 2014. Other areas formed temporary spatial clusters, including the prefectures of Mohammadia (2012), Tétouen- M’diq Fnidaq (2013), Meknes (2014), and the province of Khemissat (2014). All these cited provinces/prefectures were shown in red. Significant spatial clusters of low TB incidence were located in the east spatial regime. The provinces of Errachidia and Ouarzazate were consistently significant even at more demanding level (i.e., *P* = 0.01 instead of 0.05). Two spatial outliers (in pink) were identified, namely the prefecture of Fahs Anjra (in the north) and the province of Guelmim (in the south)

As TB incidence rate was approximatively stable from 2005 to 2014, spatial study was restricted to the last four years (i.e. 2011 to 2014). Data on recent TB cases reported in 2015, 2016 and 2017 are still not publicly available. Since box maps of TB raw rate showed only one outlier for each of the years under study (Fig. 3), we considered that rate smoothing is not necessary. Global univariate Moran’s *I* statistics, univariate LISA cluster maps, and univariate LISA significance maps of TB rates by year were all illustrated in Fig. 5. For each of the years under study, spatial clustering of high TB incidence rates was shown in the north-west part of Morocco (Global Moran’s *I* statistics were all statistically significant at 0.01 level or less). Larache and Kenitra formed high spatial clusters during the four years, Skhirate-Témara and Benslimane from 2011 to 2013, Salé from 2012 to 2014, Mohammadia in 2012, Tétouen- M’diq Fnidaq in 2013, Meknes and Khemissat in 2014, and Tanger-Assilah from 2013 to 2014 (Fig. 5). Spatial clustering of low TB incidence rates was located throughout the eastern and the southern part of the country as indicated in blue in Fig. 5. The significance level was tightened even more (to *P* = 0.01 instead of 0.05) to detect spatial clusters of low TB rates. For each of the years under consideration, the provinces of Errachidia and Ouarzazate were consistently significant even at that more demanding level. Two spatial outliers were identified, namely, Guelmim province and the prefecture of Fahs Anjra (Fig. 5).

### Univariate spatial autocorrelation, linear correlation, bivariate spatial autocorrelation, and regression model

**Fig. 6:**
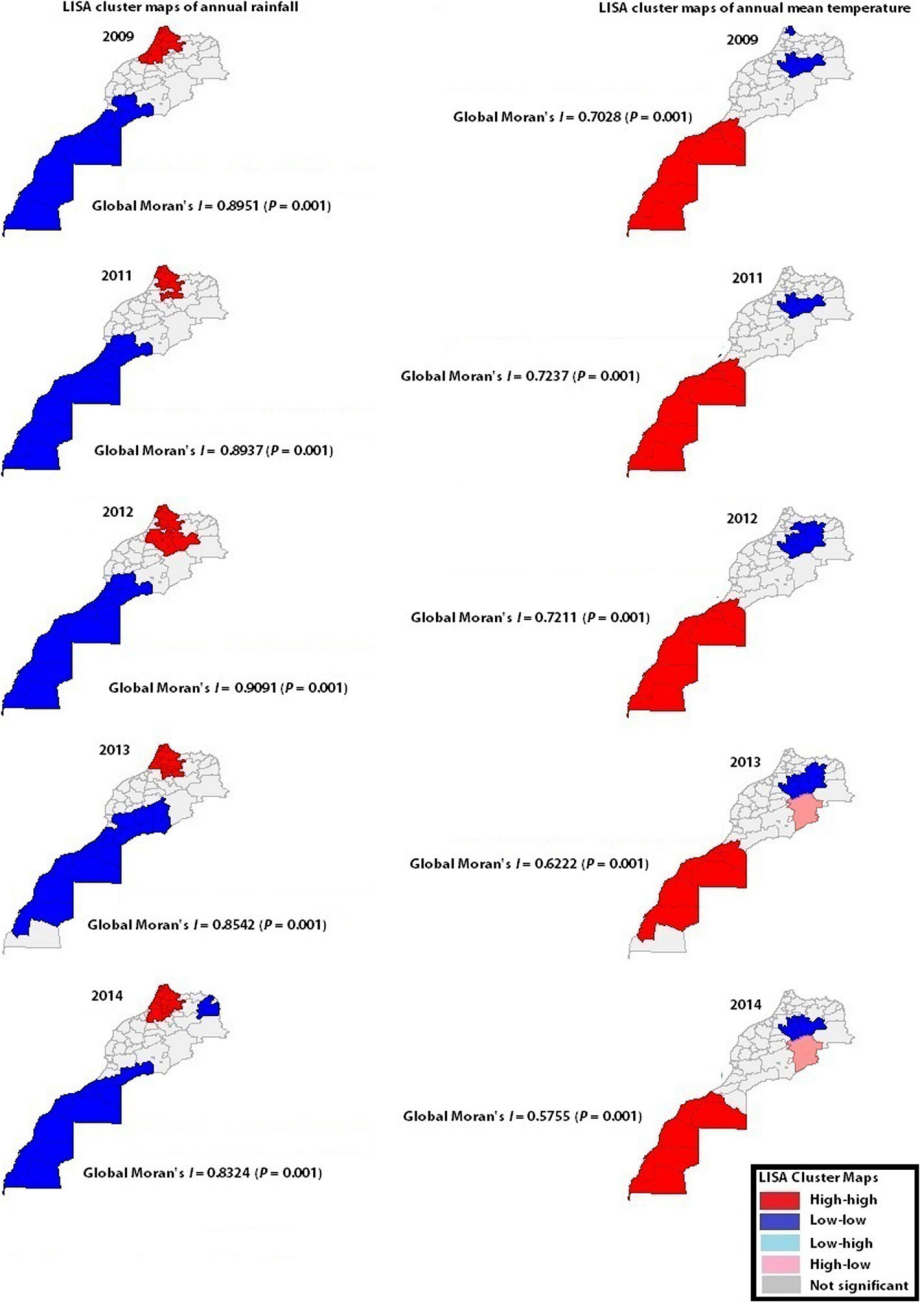
LISA cluster maps of annual rainfall and annual mean temperature, Morocco, 2009 and 2011–2014. Annual mean temperature as well as annual rainfall showed spatial clustering in all years under study. The south of Morocco has low rainfall and high temperatures. High annual rainfall clusters were shown in red (maps in left)

**Fig. 7:**
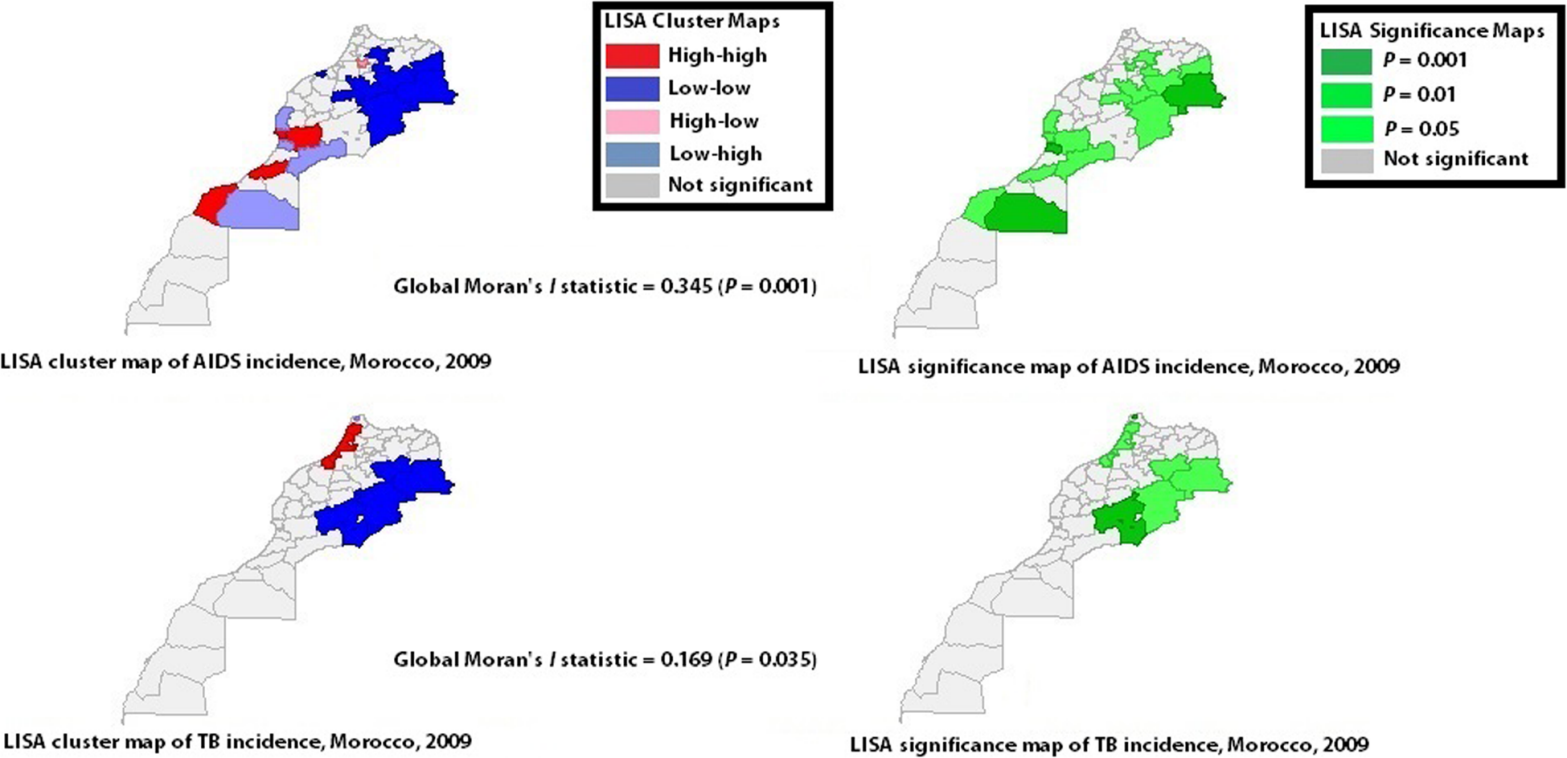
LISA cluster maps and LISA significance maps of AIDS incidence (upper maps) and TB incidence (lower maps), 2009, Morocco. Some provinces, including Taroudant, Guemim, and Layoune and some prefectures, including Inezgane Ait Melloul and Agadir Ida Outanane form spatial clusters of high AIDS incidence rates. The prefecture of Meknes formed a high-low outlier. Other provinces, including Essaouira, Tata, and Essmara and the prefecture of Chtouka Ait Baha constitute a low-high clusters. A total of 10 spatial clusters of low AIDS incidence rates were identified. However, while examining AIDS clusters at more demanding significant level (i.e., 0.01 instead of 0.05), only low incidence clusters located in the east, namely the provinces of Khenifra, Errachidia, and Figuig were identified; no high incidence cluster was seen

There was evidence of a significant spatial pattern of annual mean temperature as well as of annual rainfall in each of the years under study (Fig. 6); spatial clustering of AIDS incidence rates was also evident in 2009 (Global Moran’s *I* = 0.345; *P* = 0.001)(Table 1 and Fig. 7). The prefecture of Casablanca formed the unique cluster of population density in the country.

**Table 1:** Non-spatial and spatial correlation between TB and its potential predictors

Variable	Univariate *I* (*P*)	b (*P*)	Bivariate *I* (*P*)
Outcome (year)			
TB (2009)	0.169 (0.035)		
TB (2011)	0.229 (0.009)		
TB (2012)	0.230 (0.007)		
TB (2013)	0.389 (0.001)		
TB (2014)	0.387 (0.001)		
Covariates (year)			
Rainfall (2009)	0.895 (0.001)	0.31 (< 0.001)	0.419 (0.001)
Rainfall (2011)	0.894 (0.001)	0.196 (0.03)	0.350 (0.001)
Rainfall (2012)	0.909 (0.001)	0.203 (0.03)	0.354 (0.001)
Rainfall (2013)	0.854 (0.001)	0.380 (< 0.0001)	0.498 (0.001)
Rainfall (2014)	0.832 (0.001)	0.373 (< 0.0001)	0.491 (0.001)
Temperature (2009)	0.703 (0.001)	−0.006 (0.95)	−0.092 (0.09)
Temperature (2011)	0.724 (0.001)	−0.097 (0.29)	−0.207 (0.003)
Temperature (2012)	0.721 (0.001)	−0.087 (0.33)	−0.190 (0.001)
Temperature (2013)	0.622 (0.001)	−0.027 (0.76)	−0.091 (0.078)
Temperature (2014)	0.576 (0.001)	0.015 (0.87)	−0.065 (0.190)
Pop_Density (2009)	0.258 (0.007)	0.44 (< 0.0001)	0.225 (0.002)
Pop_Density (2011)	0.250 (0.006)	0,53 (< 0.0001)	0.246 (0.005)
Pop_Density (2012)	0.252 (0.004)	0,49 (< 0.0001)	0.250 (0.004)
Pop_Density (2013)	0.252 (0.006)	0,56 (< 0.0001)	0.257 (0.004)
Pop_Density (2014)	0.259 (0.004)	0,50 (< 0.0001)	0.182 (0.011)
AIDS (2009)	0.345 (0.001)	0.26 (0.006)	−0.139 (0.019)

*I* = Moran’s Index statistic; *P* = *P*-Value; b = Kendall’s tau

Annual rainfall, population density, and AIDS rates were strongly and positively correlated with TB, the mean annual temperature was not (all related Kendall’s tau b tests were not statistically significant) (Table 1). Annual rainfall showed spatial correlation stronger than linear correlation (Bivariate *I* > Kendall’s tau b), suggesting that this correlation is determined by geographic location. The population density showed spatial correlation weaker than linear correlation, which indicates that this correlation is partly determined by location. Table 1 also showed a statistically significant dispersion of TB rates correlated with AIDS rates (Global Moran’s *I* = − 0.139; *P* = 0.019).

Multicollinearity was seen between annual rainfall and mean annual temperature in 2009, 2011, 2012, 2013, and 2014. Kendall’s tau b were − 0.41, − 0.60, − 0.59, − 0.44, and − 0.28, respectively; all of which were statistically significant at less than 0.001 level. Multicollinearity was also seen between the population density and area (prefecture versus province) in 2009, 2011, 2012, 2013, and 2014. Kendall’s tau b were 0.59, 0.61, 0.60, 0.61, 0.61, respectively; all of which were statistically significant at less than 0.0001 level.

Taking into account both correlation and multicollinearity, we chose a regression model that includes only the annual rainfall and the spatial regimes as predictors of TB incidence rates for each of the five years under study; thus, we performed OLS regression (Table 2). Annual rainfall was consistently related to TB only in 2013 (*P* = 0.008), the spatial regime in all the years under study (*P* ≤ 0.03 were statistically significant), indicating a potential different effect of predictors across the west and the east regimes (Table 2). This led us to perform a spatial Chow test (Table 3). It was statistically significant (*P* < 0.03) for each of the years under consideration (Table 3), but annual rainfall has relatively stable effect across the east and west (*P* > 0.05) (Table 3). This suggests the influence of underlying variables that exert a different effect across the west and the east regimes.

**Table 2:** Ordinary Least Squares Regresssion of TB rates in 2009 and between 2010 and 2014. Morocco

Covariate (year)	Coefficient	*t*-Statistic	Probability
Intercept (2009)	24.179	1.944	0.057
Intercept (2011)	23.874	2.123	0.038
Intercept (2012)	22.568	1.957	0.055
Intercept (2013)	23.395	2.922	0.005
Intercept (2014)	23.863	2.186	0.033
Annual Rainfall (2009)	0.0587	1.710	0.093
Annual Rainfall (2011)	0.037	1.465	0.149
Annual Rainfall (2012)	0.051	1.691	0.096
Annual Rainfall (2013)	0.058	2.743	0.008
Annual Rainfall (2014)	0.060	1.808	0.076
Spatial regime (2009)	29.713	2.229	0.030
Spatial regime (2011)	45.149	4.240	< 0.0001
Spatial regime (2012)	40.340	3.817	< 0.001
Spatial regime (2013)	36.664	3.700	< 0.001
Spatial regime (2014)	30.152	2.474	0.016
	Value	F-statistic	*P* (F-statistic)
Adjusted R Squared (2009)	21.25%	8.691	< 0.001
Adjusted R Squared (2011)	28.72%	12.686	< 0.0001
Adjusted R Squared (2012)	26.67%	11.548	< 0.0001
Adjusted R Squared (2013)	39.53%	19.962	< 0.000001
Adjusted R Squared (2014)	32.77%	15.138	< 0.00001

**Table 3:** Spatial Regimes (West & East) Diagnostics. Chow test. 2009 and between 2010 and 2014, Morocco

Variable	DF	Value	Probability
Intercept (2009)	1	0.253	0.615
Intercept (2011)	1	5.776	0.016
Intercept (2012)	1	1.872	0.171
Intercept (2013)	1	3.030	0.082
Intercept (2014)	1	0.455	0.500
Annual Rainfall (2009)	1	0.435	0.510
Annual Rainfall (2011)	1	0.185	0.667
Annual Rainfall (2012)	1	0.096	0.757
Annual Rainfall (2013)	1	0.471	0.493
Annual Rainfall (2014)	1	3.613	0.057
Global Chow test (2009)	2	6.767	0.034
Global Chow test (2011)	2	22.603	< 0.0001
Global Chow test (2012)	2	18.348	0.0001
Global Chow test (2013)	2	18.009	0.0001
Global Chow test (2014)	2	14.359	< 0.001

Diagnostics of multicollinearity condition numbers did not suggest problems with the stability of the regression results, that may be due to multicollinearity (all numbers related to the study years were less than 8) (Table 4). The models assume normality of the errors, as indicated by the Jarque-Bera test (all *P*-values were > 0.05) (Table 4). Apart from that of the year 2009, all the *P*-values corresponding to Heteroskedasticity-related tests were small, indicating heteroscedasticity was present (*P* < 0.05) (Table 4). Diagnostics for spatial dependence for weights matrix (row-standardized weights) did not indicated the presence of spatial dependence. Lagrange Multiplier tests (lag and error tests) were not statistically significant (*P* > 0.05) (Table 4).

**Table 4:** Regression diagnosis by year

Multicollinearity condition number:					
Year	Number				
2009	5.54				
2011	4.68				
2012	4.96				
2013	3.97				
2014	7.08				
Normality of errors (Jarque-Bera test)					
Year	DF	Value	*P*-Value		
2009	2	0.522	0.770		
2011	2	0.404	0.817		
2012	2	0.471	0.790		
2013	2	2.858	0.240		
2014	2	1.289	0.525		
Heteroskedasticity coefficients:		Koenker-Bassett test		Breusch-Pagan test	
Year	DF	Value	*P*-Value)	Value	*P*-Value
2009	2	4.4506	0.108	5.338	0.069
2011	2	10.823	0.004	12.867	0.002
2012	2	9.534	0.009	11.400	0.003
2013	2	9.007	0.011	10.799	0.005
2014	2	6.340	0.042	4.311	0.116
Spatial dependance for weight matrix (row-standardized weights)					
	MI/DF	Value	*P*-Value		
Moran’s I (error) test					
2009	−0.079	−0.408	0.683		
2011	−0.046	−0.002	0.998		
2012	−0.050	−0.049	0.961		
2013	0.015	0.702	0.483		
2014	0.174	2.497	0.013		
Lagrange Multiplier (lag) test					
2009	1	0.363	0.547		
2011	1	0.062	0.804		
2012	1	0.065	0.799		
2013	1	0.442	0.506		
2014	1	3.460	0.063		
Lagrange Multiplier (error) test					
2009	1	0.727	0.394		
2011	1	0.246	0.620		
2012	1	0.292	0.589		
2013	1	0.028	0.867		
2014	1	3.564	0.059		

Unit of analysis = 59; *MI*  Moran’s I, *DF* Degree of Freedom

Given the strong heteroscedasticity and the results of the Chow test, we examined regression of TB incidence rates on covariates in the west and the east regimes, separately. Since multicollinearity was present between annual rainfall and mean annual temperature, and between area and population density for each of the studied years in both spatial regimes, we performed OLS regression of TB rates on annual rainfall and area (Tables 5 and 6). In the east, there was no evidence of spatial dependence (*P*-value related to lag tests were > 0.05) or heteroscedasticity (*P*-value related to error tests were > 0.05) (Table 7). In the west, heteroscedasticity was present only in 2012 (*P*-values related to Koenker-Bassett test and Breusch-Pagan test were 0.014 and 0.003, respectively) (Table 8); spatial dependence was not present.

**Table 5:** Ordinary Least Squares Regression of TB rates in 2009 and between 2010 and 2014. East regime. Morocco

Covariate (year)	Coefficient	t-Statistic	Probability
Intercept (2009)	30.949	2.384	0.027
Intercept (2011)	21.399	2.753	0.012
Intercept (2012)	23.721	2.677	0.014
Intercept (2013)	26.060	3.918	0.001
Intercept (2014)	36.031	3.685	0.002
Annual Rainfall (2009)	0.026	0.605	0.552
Annual Rainfall (2011)	0.041	1.990	0.060
Annual Rainfall (2012)	0.041	1.632	0.118
Annual Rainfall (2013)	0.037	1.592	0.126
Annual Rainfall (2014)	0.008	0.250	0.805
Prefecture vs province (2009)	39.323	1.166	0.257
Prefecture vs province (2011)	30.375	1.319	0.202
Prefecture vs province (2012)	35.128	1.369	0.186
Prefecture vs province (2013)	39.445	1.703	0.103
Prefecture vs province (2014)	30.400	1.202	0.243

Adjusted R Squared < 0.2 for all studied years; p (F-statistic) > 0.05 for all years except for the year 2011 for which *P* = 0.044)

**Table 6:** Ordinary Least Squares Regresssion of TB rates in 2009 and between 2010 and 2014. West regime. Morocco

Covariate (year)	Coefficient	t-Statistic	Probability
Intercept (2009)	40.299	1.703	0.099
Intercept (2011)	61.578	3.232	0.003
Intercept (2012)	46.563	2.170	0.038
Intercept (2013)	45.894	3.304	0.002
Intercept (2014)	19.455	0.700	0.489
Annual Rainfall (2009)	0.052	1.080	0.289
Annual Rainfall (2011)	0.015	0.368	0.716
Annual Rainfall (2012)	0.053	1.024	0.313
Annual Rainfall (2013)	0.058	2.047	0.049
Annual Rainfall (2014)	0.104	1.899	0.067
Prefecture vs province (2009)	41.335	2.673	0.012
Prefecture vs province (2011)	42.693	2.908	0.007
Prefecture vs province (2012)	38.846	2.724	0.010
Prefecture vs province (2013)	34.896	2.917	0.006
Prefecture vs province (2014)	31.151	2.630	0.013
	Value	F-statistic	p (F-statistic)
Adjusted R Squared (2009)	19.17%	4.914	0.014
Adjusted R Squared (2011)	16.85%	4.446	0.020
Adjusted R Squared (2012)	16.84%	4.442	0.020
Adjusted R Squared (2013)	26.67%	7.182	0.003
Adjusted R Squared (2014)	25.12%	6.704	0.004

**Table 7:** Regression diagnosis by year. East spatial regime

Multicollinearity condition number:					
Year	Number				
2009	3.64				
2011	3.31				
2012	3.20				
2013	2.56				
2014	3.66				
Normality of errors (Jarque-Bera test)					
Year	DF	Value	*P*-Value		
2009	2	7.837	0.020		
2011	2	1.250	0.535		
2012	2	0.894	0.640		
2013	2	0.441	0.802		
2014	2	1.219	0.544		
Heteroskedasticity coefficients:		Koenker-Bassett test		Breusch-Pagan test	
Year	DF	Value	*P*-Value	Value	*P*-Value
2009	2	1.952	0.377	4.112	0.128
2011	2	1.738	0.419	1.680	0.432
2012	2	1.260	0.533	1.207	0.547
2013	2	0.672	0.714	0.586	0.746
2014	2	2.591	0.274	1.389	0.499
Spatial dependance for weight matrix (row-standardized weights)					
	MI/DF	Value	*P*-Value		
Moran’s I (error) test					
2009	−0.042	0.320	0.749		
2011	0.06	1.020	0.308		
2012	0.145	1.414	0.157		
2013	0.033	0.742	0.458		
2014	0.358	2.942	0.003		
Lagrange Multiplier (lag) test					
2009	1	0.148	0.700		
2011	1	0.274	0.600		
2012	1	0.234	0.629		
2013	1	0.093	0.760		
2014	1	0.844	0.358		
Lagrange Multiplier (error) test					
2009	1	0.061	0.805		
2011	1	0.130	0.718		
2012	1	0.484	0.487		
2013	1	0.039	0.843		
2014	1	0.473	0.491		

Unit of analysis = 59; *MI* Moran’s I, *DF*  Degree of Freedom

**Table 8:** Regression diagnosis by year. West spatial regime

Multicollinearity condition number:					
Year	Number				
2009	7.00				
2011	5.66				
2012	6.62				
2013	5.01				
2014	11.08				
Normality of errors (Jarque-Bera test)					
Year	DF	Value	*P*-Value		
2009	2	1.833	0.399		
2011	2	0.721	0.697		
2012	2	2.038	0.361		
2013	2	0.276	0.802		
2014	2	0.670	0.715		
Heteroskedasticity coefficients:		Koenker-Bassett test		Breusch-Pagan test	
Year	DF	Value	*P*-Value	Value	*P*-Value
2009	2	5.123	0.036	5.123	0.077
2011	2	5.133	0.077	5.996	0.050
2012	2	8.586	0.014	11.499	0.003
2013	2	4.741	0.093	5.583	0.061
2014	2	2.710	0.258	1.794	0.408
Spatial dependance for weight matrix (row-standardized weights)					
	MI/DF	Value	*P*-Value		
Moran’s I (error) test					
2009	−0.188	−1.024	0.305		
2011	−0.121	−0.509	0.610		
2012	−0.143	−0.705	0.481		
2013	−0.073	−0.082	0.934		
2014	−0.0767	−0.166	0.868		
Lagrange Multiplier (lag) test					
2009	1	2.633	0.105		
2011	1	1.244	0.265		
2012	1	1.493	0.222		
2013	1	0.191	0.662		
2014	1	0.076	0.783		
Lagrange Multiplier (error) test					
2009	1	0.419	0.518		
2011	1	0.912	0.340		
2012	1	1.266	0.261		
2013	1	0.333	0.564		
2014	1	0.366	0.545		

Unit of analysis = 59; *MI*  Moran’s I, *DF*  Degree of Freedom

Both annual rainfall and area affect TB in the years between 2011 and 2014 and in 2009, similarly (Tables 5 and 6). This led us to incorporate AIDS rates as an additional covariate in the regression model (Table 9). In the east, annual rainfall as well as AIDS exerts a statistically significant effect on TB (respective *P*-values were 0.003 and 0.0002). In the west, only the living area was statistically significant (*P* = 0.048), suggesting that individuals living in prefectures are more exposed to TB transmission than those living in provinces (Table 9).

**Table 9:** Ordinary Least Squares Regresssion of TB rates in 2009. Morocco

Covariate (year)	Coefficient	*t*-Statistic	Probability
Spatial East Regime			
Intercept	−13.562	−1.009	0.326
Annual Rainfall	0.130	3.377	0.003
Prefecture versus province	34.599	1.444	0.165
AIDS	20.538	4.549	0.0002
Spatial West Regime			
Intercept	20.643	0.591	0.559
Annual Rainfall	0.0843	1.314	0.199
Prefecture versus province	35.565	2.059	0.048
AIDS	4.890	0.770	0.447
	Value	F-statistic	*P* (F-statistic)
Adjusted R Squared (East)	0.495	8.19 3	0.001
Adjusted R Squared (West)	0.181	3.431	0.029

East: Koenker-Bassett Statistic: 7.63 (*P* = 0.054); Jarque-Bera statistics: 0.08 (*P* = 0.96); Multicollinearity condition number: 5.5; Spatial autocorrelation of residuals: Moran’s *I*: 0.24 (*P* = 0.81); Langrange multiplier (lag): 0.09 (*P* = 0.77); Langrange multiplier (error): 0.11 (*P* = 0.74)

West: Koenker-Bassett Statistic: 7.80 (*P* = 0.19); Jarque-Bera statistics: 1.99 (*P* = 0.37); Multicollinearity condition number: 11.0; Spatial autocorrelation of residuals: Moran’s I: -0.96 (*P* = 0.34); Langrange multiplier (lag): 2.52 (*P* = 0.11); Langrange multiplier (error): 0.84 (*P* = 0.18)

## Discussion

To our knowledge, this is the first study to explore trends in TB incidence rate in Morocco, its spatial patterns and predictors, and the spatial patterns of its predictors. TB incidence rate was stable at the country level. A close look at TB incidence in the prefectures and provinces that had the highest rates was informative. Included in such group are those that form spatial clusters, including Tanger-Assilah, Tetouen-M’diq, Salé and Guelmim, and those that do not form spatial clusters, including Fez, Casablanca, Inezgane-Ait Melloul, Mohamadia, and Al Hoceima. A significant increase in TB incidence rate was seen in Tanger-Assilah. It is believed that poverty and housing conditions in some communes within this prefecture are the main risk factors of TB, and this may be subjective due to the scarcity of existing research that explores such issues in this prefecture or in others in Morocco. TB incidence rates in the prefecture of Fahs Anjra, a low-high outlier located between Tanger-Assilah and Tetouen M’ diq, were influenced by TB rates in these neighbouring prefectures. TB incidence rates in Tetouen-M’diq, Mohamadia, El Hoceima, Guelmim, and Casablanca remained stable, and this suggests that more efforts and research may be required. A high incidence cluster surrounded by low incidence clusters was observed in Guelmim Province; it was not seen in 2009 (Fig. 7). Casablanca has a population density ≥ 150 inhabitants per km^2^ [17], so have other prefectures [17]; however, only Casablanca was found to form a spatial cluster of population density in Morocco, and this was not expected. Besides, Casablanca did not form a spatial cluster of TB as it is believed. Attention has to be paid to all these previously cited areas that may require further research and new efficient strategic measures.

In Morocco, TB spatial distribution is not random. An enduring spatial clustering of high TB incidence rates was seen in the north-western part of the country, that of low TB incidence rates throughout the south and the east parts of the country. This supports our hypothesis suggesting a different effect of potential predictors on TB across the two key distinctive spatial regimes, i.e., the west and the east, that were identified in this study.

Spatial patterns of further predictors were explored. Both annual rainfall and mean annual temperature showed spatial clustering and were negatively correlated, and this was expected. The south of Morocco generally has low rainfall and high temperatures, the north of the country high rainfall and low temperatures. Previous studies carried out elsewhere [5–7] pointed to a connection between temperature and TB and to a negative correlation between rainfall and TB, and this is not consistent with the findings of this study that suggested that annual mean temperature was not correlated with TB, and that annual rainfall was positively correlated with TB. Differences in meteorological factors between countries might be one potential explanation. In our study, annual mean temperatures varied approximatively between 10 °C and 25.6 °C [data not shown], annual mean rainfall between 10.1 and 723 mm [data not shown]. In a study undertaken in China, the monthly average temperature varied between − 13.4 °C and 20.4 °C, the monthly rainfall between 0 and 195.1 mm [5]. This raises the question of whether the bacterium Mycobacterium tuberculosis can grow and spread especially well in particular conditions of temperature, humidity and probably other meteorological factors. Another question of whether there are underlying factors that may serve as an intermediary factors between meteorological conditions and TB trends is also raised. Previous ecological studies have suggested that “broad socio-economic development, rather than the success of TB control programmes, is the main determinant behind the declining trends of TB observed in many regions of the world” [18, 19]. In Morocco, inequalities in socio-economic conditions, including housing conditions, vary by province/prefecture as well as inside prefectures. Increasing rainfall may affect over-crowded dwellings and cause dwellers to be at greater risk of developing TB. This might explain annual rainfall association with TB in the east regime where the provinces of Morocco prevail, and may also explain the connection between high spatial clusters of TB and rainfall in some prefectures in the west regime. Further research that uses new scientific approaches such as mapping diseases and risk analysis at a small-area level are likely to be required in the suspected areas.

More focus on AIDS/HIV related to TB may be needed in Morocco. In a previous study, AIDS was reported to be prevalent in Agadir, Marrakesh and Casablanca [20]. A particular stakeholders’ focus may have been made on these prefectures. Our study revealed the presence of five high spatial clusters of AIDS incidence rates, all located in the west regime. Examining spatial patterns of AIDS incidence at a more demanding significant level (i.e., 0.01 instead of 0.05) yielded only low incidence clusters, formed by Khenifra, Errachidia, and Figuig, all located in the east regime. This supports our previous finding with regard to the association between TB and AIDS seen only in the east spatial regime, and may require particular decision making’s attention and further small-area studies that consider not only AIDS/HIV incidence but also socio-economic and meteorological factors as well.

This study has several strengths. It is based on reliable data, obtained from “Santé en Chiffres” files, that are representative of the population of Morocco; the Climate monitoring data, obtained from the Global Climate Monitor, was also used. Both data sources have been proven in this study to be reliable for epidemiological research. Exploring spatial clustering of TB, including spatial effect, and identifying two distinctive spatial regimes at the country level are other important strengths of this study. This study has a few limitations. Like any country-level surveillance data, some TB cases may not be reported, but they may be minor as all the physicians in the country are conscious of the importance of reporting TB cases, and TB diagnosis and treatment are free and available at the primary healthcare centres, which are the first level health facility. On the other hand, aggregate rather than individual level data were considered, which may be another common limitation of this ecological study. In spite of this, this study did yield new informative TB-related findings that may contribute to guide decision-making in Morocco and to urge to further pertinent research studies on TB.

## Conclusions

In Morocco, TB is not randomly distributed in space. Two distinctive spatial regimes that affect TB spatial clustering were identified. Planning of TB control and prevention measures/strategies that focuses on both suspected prefectures and provinces may be required and would best address this health problem. A critical need is to conduct research that considers all the herein studied risk factors and uses new scientific approaches such as geographic information systems (GIS) and risk analysis at a small-area level. More publicly available aggregated data on HIV or other health outcomes will definitely be a useful tool with regard to epidemiological research in Morocco. Questions are arisen about underlying risk factors linked to rainfall that may influence TB incidence and about association between annual rainfall and TB, and this may be of interest to be explored elsewhere.

## Additional file

Additional file 1: Multilingual abstract in the five official working languages of the United Nations. (PDF 480 kb)

## Abbreviations

AIDS: Acquired Immune Deficiency Syndrome
LISA: Local Indicators of Spatial Association
MH: Ministry of Health
*SD*: Standard Deviation
SSHHI: Service of Studies in Health and Health Information-Ministry of Health
TB: Tuberculosis
WHO: World Health Organization
