# Supplementary material for: Spatiotemporal distribution and predictors of tuberculosis incidence in Morocco
Source: Infect Dis Poverty. 2018 Jun 7;7:43. doi: 10.1186/s40249-018-0429-0 (PMC5991459; doi:10.1186/s40249-018-0429-0)
Supplement: Supplementary file 1 — Multilingual abstract in the five official working languages of the United Nations. (PDF 480 kb) [file 40249_2018_429_MOESM1_ESM.pdf]

Translation of the abstract into the five official working languages of the United Nations

التوزيع المكاني والزماني ومؤشرات مرض السل في المغرب

مينا صادق وجمال الدين بورقادي

الملخص
المعلومات الأساسية:يعد السل أحد أهم المشكلات الصحية الرئيسية في المغرب. تهدف هذه الدراسة إلى تناول اتجاهات مرض السل في المغرب وتحديد التكتلات الجغرافية للسل وومؤشرات هذا المرض.
الأسلوب: استخدمت بيانات أجهزة المراقبة على مستوى الدولة. تم استخدام اختبار ارتباط كندال لدراسة اتجاهات مرض السل وإجراء تحليل استكشافي للبيانات المكانية من أجل تقييم الأنماط العالمية والمحلية للعلاقة الذاتية المكانية في معدلات السل (مؤشر موران الأول والمؤشر المحلي للرابطة المكانية) على مستوى المقاطعة/الإقليم. ووضعت المتغيرات المختلفة في الحسبان بما في ذلك العيش في الحضر مقابل العيش في الريف، وهطول الأمطار السنوي، ودرجة الحرارة المتوسطة السنوية، والكثافة السكانية، والإصابة بالإيدز. وتم إجراء انحدار المربعات الصغرى العادية بناء على ذلك وتقييم كلاً من التبعية المكانية واختلاف التباين.
النتائج: لوحظ انخفاض في معدل الإصابة بالسل بين عامي 1995 و 2014 (معامل ارتباط كندال للرتب ب= 0.72 P- القيمة الاحتمالية <0.0001). وفي حين أن الفترة بين عامي 2005 و 2014 (10 سنوات الماضية) وضعت في الاعتبار فقد ظلت معدلات السل مستقرة وارتفعت لتصل إلى 84 لكل 100000 نسمة سنويا (95٪مجال الثقة= 83.7 - 84.3). وظهرت أعلى معدلات الإصابة في طنجة - أصيلة، وفاس، وتطوان - عمالة المضيق الفنيدق، وإنزكان، وآيت ملول، والدار البيضاء. بين عامي 2005 و 2014، بينما كان معدل الإصابة بالسل مستقراً في فاس (P = 0.500)، كان في تطوان-عمالة المضيق الفنيدق (P = 0.300)، الدار البيضاء (P = 0.500)، المحمدية (P = 0.146)، الحسيمة (P = 0.364)، و كلميم (P = 0.242)، لوحظت زيادة في معدلات الإصابة بالسل في طنجة - أصيلة (معامل ارتباط كندال ب = 0.49؛ P = 0.023) وانخفض في سلا (معامل ارتباط كندال ب = 0.54؛ P = 0.014) و إنزكان-أيت ملول (معامل ارتباط كندال ب = 0،67؛ P = 0.0023). يتجمع السل بقوة في أماكن (P - قيم دليل موران <0.01). وتم تحديد نظامين مكانيين متميزين يؤثران على التكتل الجغرافي لمرض السل (الشرق والغرب). في الشرق، كان لكل من هطول الأمطار السنوي (P = 0.003) والإيدز(P = 0.0002) تأثير ذو دلالة إحصائية على معدلات السل. في الغرب، كانت مناطق العيش فقط (المقاطعة في مقابل الإقليم) مرتبطة بمعدلات السل (P = 0.048).
النتائج: توافرت معلومات جديدة عن حالات الإصابة بالسل ومؤشرات المرض أثناء عملية صنع القرار ومن أجل إجراء المزيد من الأبحاث ذات الصلة. وقد تكون دراسة العلاقة بين هطول الأمطار السنوي ومرض السل أمراً مهماً في أماكن أخرى.

Translated from English version into Arabic by Yousra Fakhrey, through

TRANSLATORS
WITHOUT BORDERS

摩洛哥结核病时空分布及发病率预测因子

Mina Sadeq and Jamal Eddine Bourkadi

摘要
引言：结核病（TB）是摩洛哥主要的健康问题之一。本研究旨在研究摩洛哥 TB 的发展趋势，并确定 TB 的空间聚集性及其相关预测因子。
方法：本研究使用国家级的 TB 监测数据，采用肯德尔的相关试验进行趋势研究和探索性的空间数据分析，以评估在地区/省一级的结核发病率（Moran's *I* 和空间协会的局部指标[LISA]）的全部和当地的空间自相关性模式。协变量包括居住地（省/市）、年降雨量、年平均温度、

人口密度和艾滋病发病率。本研究还进行了最小二乘回归研究，评估了其空间依赖性和异方差性。
结果：1995 年至 2014 年间，摩洛哥 TB 发病率有所下降（Kendall’s tau b = -0.72； P-value < 0.0001）。但 2005 年至 2014 年间（10 年），TB 发病率基本稳定，每年为 80/100,000（95% CI = 83.7 – 84.3）。发病率最高的地区为丹吉尔-阿西拉、菲兹、蒂图恩-米德克·法尼达克、奈兹甘·艾特·梅勒尔和卡萨布兰卡。从 2005 年到 2014 年， 菲斯(P = 0.500)、蒂图恩-米德克·法尼达克(P = 0.300)、卡萨布兰卡(P = 0.500)、穆罕默迪耶(P = 0.146)、阿尔·胡塞马(P = 0.364)和盖勒敏(P = 0.242)的 TB 发病率较稳定，而丹吉尔-阿西拉(Kendall’s tau = 0.49; P = 0.023)的 TB 发病率上升，萨累(Kendall’s tau b = -0,54; P = 0.014)和 Inezgane-Ait Melloul (Kendall’s tau b = -0,67; P = 0.0023)的 TB 发病率下降。TB 具有很强的空间聚集性(Moran's I 的 P < 0.01)。本研究确定了 TB 空间聚类的两个不同的空间因素（东部、西部）。在摩洛哥东部，年降雨量(P = 0.003)和艾滋病(P = 0.0002)对 TB 发病率的影响具有统计学意义。在西方，只有居住地区(州与省)与 TB 发病率相关(P = 0.048)。
结论：本研究为制定 TB 相关决策和开展进一步研究提供了发病率及其相关预测因子的新信息。年降雨量和 TB 之间的关联性值得进一步探索。

Translated from English version into Chinese by Xin-Yu Feng, edited by Jin Chen, through

CHINESE CENTER FOR DISEASE CONTROL AND PREVENTION
NATIONAL INSTITUTE OF PARASITIC DISEASES

Distribution spatiotemporelle et prédicteurs de l’incidence de la tuberculose au Maroc

Mina Sadeq et Jamal Eddine Bourkadi

Résumé

Contexte : La tuberculose est un problème de santé publique majeur au Maroc. La présente étude avait pour but d’examiner ses tendances et d’identifier les concentrations spatiales de cas et les prédicteurs qui lui sont associés.
Méthode : Nous avons exploité les données de surveillance à l’échelle nationale. Le test de corrélation de Kendall a été utilisé pour examiner les tendances et nous avons procédé à une analyse exploratoire des données spatiales afin d’évaluer les schémas globaux et locaux d’autocorrélation spatiale des taux de tuberculose (indice de Moran et indicateur local d’association spatiale [LISA]) au niveau des préfectures et des provinces. Nous avons contrôlé l’influence des covariables, notamment du fait de vivre dans une préfecture ou une province, de la pluviométrie annuelle, de la température moyenne annuelle, de la densité de population et de l’incidence du SIDA. Une régression aux moindres carrés ordinaire a donc été réalisée et la dépendance spatiale ainsi que l’hétéroscédasticité ont été évaluées.
Résultats : On observe une baisse de l’incidence de la tuberculose entre 1995 et 2014 (tau-b de Kendall = -0,72 ; P < 0,0001). Toutefois, sur la période de 2005 à 2014 (10 dernières années), le taux est resté stable au niveau relativement élevé de 84 cas pour 100 000 habitants par an (IC à 95 % de 83,7 à 84,3). Les plus forts taux d’incidence ont été observés à Tanger-Assilah, Fès, Tétouan-M’diq Fnidaq, Inezgane Aït Melleoul et Casablanca. De 2005 à 2014, alors que l’incidence de la tuberculose était stable à Fès (P = 0,500), Tétouan-M’diq Fnidaq (P = 0,300), Casablanca

(P = 0,500), Mohammédia (P = 0,146), Al Hoceima (P = 0,364) et Guelmim (P = 0,242), elle a augmenté à Tanger-Assilah (tau de Kendall = 0,49 ; P = 0,023) et baissé à Salé (tau-b de Kendall = -0,54 ; P = 0,014) et Inezgane-Aït Melloul (tau-b de Kendall = -0,67 ; P = 0,0023). Les cas de tuberculose sont fortement regroupés dans l'espace (valeurs de P de l'indice de Moran < 0,01). Deux régimes spatiaux distincts influant sur la concentration spatiale des cas (Est et Ouest) ont été identifiés. Dans l'Est, les précipitations annuelles (P = 0,003) et le SIDA (P = 0,0002) ont un effet statistiquement significatif sur le taux de tuberculose. Dans l'est, seul le lieu de résidence (préfecture ou province) était associé au taux de tuberculose (P = 0,048).

Conclusions : Nous avons recueilli de nouvelles informations sur l'incidence de la tuberculose et ses facteurs prédictifs, à l'appui des prises de décisions et des futures recherches en la matière. Il pourrait être intéressant d'explorer, dans un autre cadre, l'association entre pluviométrie annuelle et tuberculose.

Translated from English version into French by Liudmila Tomanek and Natalia Potashnik, through

TRANSLATORS WITHOUT BORDERS

Пространственно-временное распространение и прогностические факторы заболеваемости туберкулёзом в Марокко

Мина Садик и Джамаль Эддин Буркади

Аннотация

Краткое описание. Туберкулёз (ТБ) представляет собой значительную проблему для здравоохранения в Марокко. Данное исследование нацелено на анализ динамики ТБ в Марокко, а также на выявление пространственной кластеризации ТБ и прогностических факторов заболевания.

Метод. Были использованы данные наблюдения на страновом уровне. Для изучения динамики был использован коэффициент ранговой корреляции Кендалла, тогда как для оценки глобальных и местных моделей пространственной автокорреляции по уровню заболеваемости ТБ был проведён разведывательный анализ пространственных данных (индекс Морана I и местный индикатор пространственных ассоциаций [LISA]) на уровне префектур/провинций. В наблюдение вошли такие коварианты, как проживание в префектуре в сравнении с проживанием в провинции, годовое количество осадков, среднегодовая температура воздуха, плотность населения, а также заболеваемость СПИДом. Таким образом, была выполнена регрессия по методу наименьших квадратов, а также произведена оценка пространственной зависимости и гетероскедастичности.

Результаты. С 1995 года по 2014 год наблюдалось снижение уровня заболеваемости ТБ (тау Кендалла b = -0,72 P-значение < 0,0001). Однако изучение периода с 2005 года по 2014 год (последние 10 лет) показало, что уровень ТБ оставался стабильным и достаточно высоким с величиной 84 случаев на 100000 человек в год (95 % CI = 83,7 – 84,3). Наиболее высокий

уровень заболеваемости наблюдался в Тангер-Ассилахе, Фезе, Тетуэн-Мдик Финидаке, Инезгане Аит Меллуле и Касабланке. С 2005 года по 2014 год уровень заболеваемости ТБ оставался стабильным в Фезе (*P* = 0,500), Тетуэн-Мдик Финидаке (*P* = 0,300), Касабланке (*P* = 0,500), Мохаммадии (*P* = 0,146), Эль-Хосейме (*P* = 0,364) и Гулимине (*P* = 0,242). В то же время наблюдалось повышение уровня ТБ в Тангер-Ассилахе (тау Кендалла = 0,49; *P* = 0,023) и снижение в Сале (тау Кендалла b = -0,54 ; *P* = 0,014) и Инезгане-Аит Меллуле (тау Кендалла b = -0,67; *P* = 0,0023). Наблюдается высокая пространственная сосредоточенность ТБ (*P*-значения индекса Морана I < 0,01). Были выявлены два различных пространственных режима, воздействующих на пространственную сосредоточенность ТБ (восток и запад). На востоке годовой уровень осадков (*P* = 0,003) и СПИД (*P* = 0,0002) оказывают статистически значимое влияние на уровень ТБ. На западе только район проживания (префектура в сравнении с провинцией) влияли на уровень заболеваемости ТБ (*P* = 0,048).
Выводы. Была предоставлена новая информация по заболеваемости ТБ и прогностическим факторам заболеваемости ТБ для принятия решений и дальнейших исследований в этом направлении. Связь между годовым количеством осадков и ТБ может представлять интерес для исследований в других районах.

Translated from English version into Russian by Natalia Potashnik, through

TRANSLATORS
WITHOUT BORDERS

Distribución Espacio-temporal e Indicadores de Frecuencia de Tuberculosis en Marruecos.

Mina Sadeq y Jamal Eddine Bourkadi

Resumen
Contexto: La tuberculosis (TB) es un gran problema sanitario en Marruecos. Este estudio tiene como objetivo examinar tendencias de TB en Marruecos e identificar agrupaciones territoriales de TB, así como también indicadores asociados a la TB.
Método: Se utilizó información de vigilancia a nivel nacional. La prueba de correlación de Kendall se utilizó para examinar tendencias y se realizó un análisis exploratorio de información espacial para determinar los patrones de auto-correlación espacial en índices de TB globales y locales (la I de Moran y el indicador local de asociación espacial [LISA, por sus siglas en inglés]) a nivel provincial/de prefectura. Se controlaron las covariables, incluidas la vida en una prefectura versus la vida en una provincia, el nivel de precipitación anual, la temperatura promedio anual, la densidad de población y la incidencia del SIDA. Se realizó entonces una regresión de mínimos cuadrados ordinarios y se evaluaron tanto la dependencia espacial como la heterocedasticidad.
Resultados: Se observó una disminución del índice de incidencia de la TB entre 1995 y 2014 (la tau b de Kendall = -0,72 *P*-valor < 0,0001). Sin embargo, si bien se consideró el período entre 2005 y 2014 (los últimos 10 años), el índice de TB permaneció estable y tan alto como 84 cada 100 000 habitantes por año (95 % *CI* = 83,7 – 84,3). Los más altos índices de incidencia se observaron en

Tanger-Assilah, Fez, Tetouen-M'diq Fnidaq, Inezgane Ait Melleoul, y Casablanca. Desde 2005 hasta 2014, si bien el índice de incidencias de TB se mantuvo estable en Fez (P = 0,500), Tetouen-M'diq Fnidaq (P = 0,300), Casablanca (P = 0,500), Mohammadia (P = 0,146), Al Hoceima (P = 0,364), y Guelmim (P = 0,242), se observó un incremento del índice en Tanger-Assilah (tau de Kendall = 0,49; P = 0,023) y una disminución en Salé (tau be de Kendall = -0,54 ; P = 0,014) y Inezgane-Ait Melloul (tau be de Kendall = -0,67 ; P = 0,0023). La TB está fuertemente agrupada en el espacio (P-Valores de la I de Moran $<$ 0,01). Se identificaron dos regímenes distintos que afectan la agrupación espacial de la TB (Este y Oeste). En el Este, tanto el nivel de precipitación anual (P = 0,003) como el SIDA (P = 0,0002) ejercen un efecto estatísticamente significante en el índice de la TB. En el Oeste, solamente el área habitable (prefectura versus provincia) se asoció con el índice de la TB (P = 0,048).
Conclusiones: Se proporcionó nueva información sobre la incidencia de la TB y los indicadores relacionados a la TB para la toma de decisiones y la posterior investigación pertinente. La asociación entre el nivel de precipitación anual y la TB puede ser interesante para explorarse en otro lugar.

Translated from English version into Spanish by Victoria Bañales, through
